# A case series measuring campus and clinic level factors during implementation of a sexual violence prevention intervention in campus health and counseling centers: does environment matter?

**DOI:** 10.1186/s43058-023-00467-7

**Published:** 2023-07-31

**Authors:** Jocelyn C. Anderson, Gabrielle Peruggia, Summer Miller-Walfish, Janine Talis, Carmen Burrell, Micaela Hayes, Elizabeth Miller

**Affiliations:** 1 Ross and Carol, Nese College of Nursing, The Pennsylvania State University, 201 Nursing Sciences Building, PA 16802 University Park, USA; 2 School of Medicine, University of Pittsburgh, 120 Lytton Ave, Pittsburgh, PA 15213 USA; 3 School of Medicine, West Virginia University, 1 Medical Center Drive, P.O. Box 9149, Morgantown, WV 26506 USA; 4 University Health Services, The Pennsylvania State University, University Park, PA 16802 USA

**Keywords:** Student health services, College health, Patient-provider communication, Sexual violence intervention

## Abstract

**Objective:**

This study examined campus and clinic factors that may influence likelihood of implementing sexual violence (SV) prevention for college students seeking care in campus health and counseling centers.

**Methods:**

Campus-, clinic-, and student-level data were collected from both intervention and control campuses as part of a 28-campus cluster randomized controlled trial. A case series exploratory data analysis assessed differences in the implementation of an SV prevention intervention by campus characteristics.

**Results:**

All large schools were in the top quartile for reporting positive prevention policies regarding SV. At the clinic level, the presence of SV protocols and procedures varied widely with no clear correlation with school size. Students at intervention schools where providers received instruction and tools to facilitate these discussions reported more discussions with providers about SV. Only school size appeared to be associated with positive SV policies on campus and student-reported receipt of SV prevention intervention. Large schools performed well on campus-level policies, yet students reported some of the lowest levels of intervention receipt in the clinics at these larger schools.

**Implications:**

Consistency between campus and clinic environments and implementation of the intervention was not observed. Our findings suggest that high performance regarding SV policy and prevention on a campus do not necessarily translate to implementation of appropriate SV prevention and care for students seeking care on campus, including assessments, resources, referrals, and services.

**Trial registration:**

NCT registration: NCT02355470

**Supplementary Information:**

The online version contains supplementary material available at 10.1186/s43058-023-00467-7.

Contributions to the literature

- This case series analysis found some patterns between school size, campus-level policies, and performance in delivering a campus health center-based sexual violence intervention.
- Small schools delivered the one-on-one intervention to students more consistently, while large schools reported more formal SV policies, procedures, and resources.
- Research is needed to determine if these patterns are evident across larger datasets and to determine strategies to improve intervention implementation.

## Introduction

Sexual violence (SV) and intimate partner violence (IPV) are complex, common, and often co-occurring public health issues facing colleges and universities [1, 2]. Sexual violence—inclusive of rape, sexual assault, online and in person sexual harassment, and other sexual contacts or attempted contact without consent—occurs with alarming frequency on campuses [2]. One large multicampus study found approximately 1 in 5 women, 1 in 20 men, and 1 in 4 gender minority students reported SV during their college career [2].

Health care systems have been identified as key locations for providing prevention efforts and support for survivors of both IPV as well as SV [3, 4]. While survivors often do not seek care specifically for violence, the likelihood that they will seek care for the myriad of health concerns resulting from violence is high [3]. Interventions in healthcare settings are effective in increasing patients’ understanding of SV and available resources [5, 6]. Thus, campus health centers are ideal settings to engage students regarding SV prevention and supportive care.

Addressing violence as a public health issue requires a multi-level approach [4, 7]. Although emphasis is placed on training health care providers to implement clinic-based interventions, such interventions are influenced by policies and other programs and services available to patients that are beyond the control of providers themselves (i.e., clinic protocols, limited access to victim services) and often external to the health center itself (i.e., local policies, resources for SV prevention, response of investigators) [8, 9]. Interventions situated within campus health centers operate within the context of policies and prevention efforts across the campus and community [10]. Implementation of trauma-sensitive practices and policies by campus security, provision of training and support for faculty, and institution of campus-wide policies that support survivors during an investigation or adjudication process are outside the control of campus health. Campus-level policies and practices may not directly impact the healthcare system or provider’s ability to implement trauma-sensitive interventions in their own practice, yet without structural supports, it may be challenging for students to actually receive trauma-sensitive services and connect to resources [8].

A quality improvement (QI) tool was used as part of a campus health center SV intervention, intended to guide health centers in implementing policies and protocols to facilitate connecting students to appropriate supports and services. Such tools are used in healthcare settings to evaluate practice initiatives, identify areas in which a practice can be improved, and implement changes [11]. By identifying specific protocols, policies, and practices to support individuals who have experienced SV and measuring these over time, campus stakeholders can evaluate facilitators for SV prevention interventions at the campus and clinic levels. This case series used data collected about SV on campuses and within the campus health center to explore potential facilitators to strengthen implementation of a campus health center SV intervention.

## Methods

### Overview

Data were collected as part of a 28 site, cluster randomized controlled trial that tested the GIFTSS intervention, a campus health center provider-delivered SV prevention intervention [12]. As part of the evaluation for the study, campus- and clinic-level data related to alcohol and SV prevention and response were collected. This case series uses available campus- and clinic-level data from the 28 sites to assess for patterns in implementation of the GIFTSS intervention. Study procedures were approved by the University of Pittsburgh Institutional Review Board.

### Data collection

#### Campus-level data: environmental scan

Environmental scan data were collected by trained research assistants. Research assistants collected data from multiple sources including structured telephone interviews with campus staff members (e.g., student affairs, campus health) and publicly available information (e.g., online resources, policies), then entered information into a secure online database [13, 14]. Environmental scans were collected prior to intervention implementation and were collected for all 28 sites (control and intervention). Domains included campus SV data, collection and reporting, prevention programming, community partnerships, SV investigation, accommodations for students who have experienced violence, and policies/procedures (Online Supplement A). While this evaluation was part of a larger RCT designed to determine effectiveness, and not purely implementation focused, these domains align clearly with those of the Center for Implementation Research’s Outer Setting—external policies and incentives, peer pressure, and patient needs and resources [15].

#### Clinic-level data: quality improvement tool

A QI tool was modified from prior work in family planning and school-based health and piloted with campus health providers prior to the study [5, 6]. Clinic-level data were collected prior to implementation and 6 months later for each of the intervention sites (*n* = 12 completed baseline,* n*= 10 completed both timepoints). Data were collected via forms mailed to clinic administrators and entered into the study database by research assistants [13, 14]. Key domains covered in this tool included policies/procedures, assessment, documentation, interventions and referrals, community partnerships, data collection, and staff training/support (Online Supplement B). Whereas the campus environment aligned with the Outer Setting construct, the immediate clinic environment aligns with the inner setting—including compatibility, relative priority, organizational incentive and rewards, available resources, and goals and feedback.

#### Student-reported data: exit survey

Student-level data were collected through student participants completed computer-based surveys following their initial clinic visit. Students were recruited from 26 distinct campuses using in-person, email, and text message methods based on clinic structure, function, and policy. In total, 2789 students were screened for eligibility, 2486 were eligible and consented to participate, and 2291 ultimately completed the baseline survey measure and were included in the study. In the parent trial, students were surveyed at four timepoints: baseline (prior to their clinic visit), exit (immediately after their clinic visit), T2 (4 months after their visit), and T3 (1 year after their visit). Specific to this analysis, during the exit survey, students were asked questions regarding aspects of the SV intervention that were received during their visit. To assess whether students received the educational safety card, they were asked: “Today, did your counselor or student health provider give you one of these palm-sized cards (pictured below)?”^1^^*^ To determine if their provider discussed SV/IPV with them during the visit, a series of eight questions were asked such as “Today, did your counselor or student health center provider talk to you about local and national resources available for you or a friend affected by sexual assault or intimate partner violence;” “Today, did your counselor or student health center provider talk to you about having sex when you don’t want to?” Students were also asked if they had disclosed SV/IPV to their provider during their visit. Additional details on student recruitment and data collection methods are available elsewhere [12, 16].

### Data analysis

Exploratory analyses were used to examine differences in intervention delivery by campus characteristics such as undergraduate university enrollment, public university (yes or no), religious affiliation, and campus location (small town, rural, or urban) (Table 1). Campus-level, clinic-level, and student-level data were analyzed using proportions of outcomes at each site to summarize responses. All demographic, environmental scan, QI, and exit survey data were visualized individually first and then combined for comparative analysis (Table 2). As the overall unit of comparison was the campus, and our total sample consisted of 28 total sites, we did not conduct formal statistical analyses, treating these data as a case series of multiple data sources.

**Table 1:** Campus level demographics

	Control (*n* = 16)	Intervention *(n* = 12)
	*% (n)*	*% (n)*
Undergraduate enrollment		
Small (< 5000)	75 (12)	66.7 (8)
Medium (5000–15,000)	18.8 (3)	16.7 (2)
Large (>15,000)	6.3 (1)	16.7 (2)
Public university		
Yes	66.7 (8)	56.3 (9)
No	33.3 (4)	43.8 (7)
Religious affiliation		
Yes	25 (4)	8.3 (1)
No	75 (12)	91.7 (11)
Campus location		
Small town/rural	18.8 (3)	8.3 (1)
Suburban	62.5 (10)	75 (9)
Urban	18.8 (3)	16.7 (2)

Rurality was determined using the campus ZIP code and 2010 Rural-Urban commuting area (RUCA) codes data available from: https://www.ers.usda.gov/data-products/rural-urban-commuting-area-codes.aspx

**Table 2:** Summary of campus- and clinic-level data

Site demographics					Campus-level data *N* = 49 questions total		Clinic-level data *N* = 89 questions total		Student-level data^a^			
#	School size	Recruitment from counseling and/or health center	Religious affiliation	Location	# Yes	# No	# Yes, at baseline	# Improved at 6 month follow-up	Received intervention as designed	Received no intervention	Disclosed SV/IPV at visit	Provider talked about SV/IPV
					*% (n)*	*% (n)*	*% (n)*	*% (n)*	*% (n)*	*% (n)*	*% (n)*	*% (n)*
Intervention schools												
1	Medium	Health	No	Urban	53.06 (26)	**18.37 (9)**	**13.48 (12)**	*15.73 (14)*	62.24 (122)	30.10 (59)	5.61 (11)	*22.44 (44)*
2	Small	Health and counseling	No	Rural	**46.94 (23)**	**22.45 (11)**	**31.46 (28)**	*24.71 (22)*	52.21 (71)	32.35 (44)	8.83(12)	18.38 (25)
3	Medium	Health	No	Urban	*61.22 (30)*	10.20 (5)	*67.42 (60)*	10.11 (8)	**7.75 (10)**	**74.42 (96)**	**1.55(2)**	**2.33 (3)**
4	Small	Counseling	No	Suburban	*57.14 (28)*	*8.16 (4)*	44.94 (40)	14.61 (13)	24 (6)	52 (13)	*32(8)*	*24 (6)*
5	Small	Health and counseling	Yes	Urban	*63.27 (31)*	*8.16 (4)*	*46.07 (41)*	**6.74 (6)**	75.38 (49)	12.30 (8)	*30.77 (20)*	*64.61 (42)*
6	Small	Health	No	Suburban	**44.90 (22)**	14.29 (7)	37.09 (33)	**6.74 (6)**	*92.59 (25)*	*7.41 (2)*	7.41(2)	*81.48 (22)*
7	Large	Health	No	Urban	*57.14 (28)*	14.29 (7)	*67.42 (60)*	*15.73 (14)*	**13.27 (15)**	**80.53 (91)**	**0.89(1)**	5.31 (6)
8	Small	Health and counseling	No	Urban	55.10 (27)	16.33 (8)	33.71 (30)	10.11 (8)	*87.69 (57)*	7.69 (5)	10.77(7)	*70.77 (46)*
9	Small	Health	No	Urban	**42.86 (21)**	**26.53 (13)**	**31.46 (28)**	*22.47 (20)*	25 (1)	**75 (3)**	**0**	*25 (1)*
10	Large	Health	No	Urban	*59.18 (29)*	10.20 (5)	**29.21 (26)**	**3.37 (3)**	22.43 (46)	55.61 (114)	4.88(10)	12.20 (25)
11	Small	Health	No	Urban	53.06 (26)	16.33 (8)	35.96 (32)	b	**0**	*0*	*100 (1)*	**0**
12	Small	Counseling	No	Urban	*57.14 (28)*	12.25 (6)	**31.46 (28)**	b	50 (1)	*0*	*50 (1)*	*100 (2)*
Control schools												
13	Medium	Health	No	Rural	55.10 (27)	16.37 (8)					**2.67 (5)**	4.81 (9)
14	Small	Health and counseling	No	Urban	**40.82 (20)**	**26.57 (14)**					8.51 (4)	8.51 (4)
15	Medium	Health	Yes	Urban	51.02 (25)	**20.40 (10)**					**2.76 (4)**	**1.38 (2)**
16	Small	Counseling	No	Urban	53.06 (26)	12.24 (6)					*37.03 (10)*	11.11 (3)
17	Medium	Health	No	Suburban	55.10 (27)	*8.16 (4)*					**2.92 (5)**	6.43 (11)
18	Large	Health	No	Urban	*59.18 (29)*	12.24 (6)					3.33 (2)	**3.33 (7)**
19	Small	Health and counseling	No	Urban	**32.65 (16)**	*6.12 (3)*					*18.60 (8)*	**0**
20	Small	Health and counseling	Yes	Urban	**44.90 (22)**	**22.45 (11)**					*16.67 (4)*	20.83 (5)
21	Medium	Health	No	Urban	55.10 (27)	*8.16 (4)*					7.5(9)	15.83 (19)
22	Small	Health	Yes	Suburban	*57.14 (28)*	*6.12 (3)*					**0**	4.76 (1)
23	Small	Health and counseling	No	Urban	**46.94 (23)**	*8.16 (4)*					4.55 (1)	9.09 (2)
24	Small	Health and counseling	No	Urban	55.10 (27)	14.29 (7)					*16.67 (7)*	9.52 (4)
25	Small	Health and counseling	No	Rural	55.10 (27)	12.24 (6)					11.11 (1)	**0**
26	Small	Health and counseling	Yes	Rural	*61.22 (30)*	10.20 (5)					4.65 (2)	**2.33 (1)**
27	Small	Health and counseling	Yes	Suburban	*57.14 (28)*	14.29 (7)					12.5 (3)	20.83 (5)
28	Small	Health	No	Urban	51.02 (25)	**18.37 (9)**					**0**	**0**

Italicized text cells note the top quartile in this performance indicator. Bold text cells note the lowest quartile in this performance indicator

Per RCT study design, clinic-level quality improvement data not collected from control sites and control sites students did not receive intervention

^a^Each campus total *N* varied based on recruitment (range 1–210)

^b^Campus did not provide a 6-month follow-up clinic-level data

#### Campus-level data: environmental scan data

Responses from each site to each of the items in the environmental scan were analyzed in Excel. Each site had the opportunity to answer each item with “yes,” “no,” or “not applicable.” Only items asked of all schools were included; some schools left items blank. For analysis, the total number of yes and no responses were calculated.

#### Clinic-level data: quality improvement tool

QI data were coded similarly to environmental scan data. Proportions were obtained for sites that indicated having a resource or policy at baseline (e.g., “yes” at baseline) and those that showed improvement from baseline to follow up (e.g., centers that went from a “no” in the baseline data to a “yes” at 6-month follow-up) (Table 2).

#### Student-reported outcome data: exit survey data

Intensity scores were assigned to students based on whether the student reporting receiving the intervention as designed [16]. An intensity score of 1 indicated that the student received both intervention components: (1) the intervention card and (2) discussion of SV/IPV with their provider. An intensity score of 0.5 was assigned to those who received only one component of the intervention, either discussion with their provider or having received the card. An intensity score of 0 was attributed to those who did not receive the intervention. Percentages of students with each intensity score were then calculated by school for comparisons. Scores and proportions were calculated in SAS [17].

## Results

### Campus-level data: environmental scan

In total, 49 questions were included in this analysis from the environmental scan. Total number of “yes” responses ranged from 16 to 31 with a median of 27 (IQR: 24, 28). The top quartile of schools with affirmative responses to the environmental scan data, included all three large universities, one medium, and six small schools—four of those six were religiously affiliated, and one was a branch campus of a large university. The bottom quartile included seven small schools, four of which were branch campuses of larger institutions, and one of which was religiously affiliated.

Nearly all (*n* = 25, 89.3%) schools reported having a task force or other group that meets regularly regarding SV to coordinate prevention or response. However, only 17 of those 25 (68%) reported that one of their responsibilities was to review the campus policies regarding SV. Similarly, almost all schools (*n* = 26, 92.9%) reported that their institution’s Clery Act report was *easily* accessible to them. After being asked to review the most recent year’s Clery Act report data, only 8 (29.6%) reported they believed the information was an accurate representation of SV prevalence on their campus. Over half of schools (*n* = 16, 57.14%) reported that their campus had completed a climate survey regarding SV in the past 3 years. One school reported not having SV prevention education available for new students, with 25 schools (92.6%) stating that SV prevention training was mandatory for students.

All but one (*n* = 27, 96.4%) reported they felt their institution provided clear reporting options for students who have experienced SV. Respondents also noted they believed students knew who to talk to if they want to anonymously report SV (*n* = 21, 75%) but were less certain students were aware of mandatory reporting procedures with 11 (39.3%) stating students understood who on campus was required to report SV. All 28 schools reported a designated individual who was responsible for informing students of their rights and responsibilities during an investigation; less than half of schools (*n* = 13, 46.4%) had dedicated full-time Title IX coordinators.

### Clinic-level data: quality improvement tool

Of the 10 sites with reported QI data at both baseline and follow-up, sites varied widely regarding protocols and procedures within their health center. Of the 89 items, the median number of items endorsed affirmatively at baseline was 31 (range: 12–60). All clinics reported improvement in some items with the median number of improvements being 11 (range: 3–22); conversely, clinics also reported areas in which they had a protocol or procedure in place at baseline and did not at follow-up (median: 5, range: 1–18).

Specific items in which clinics showed improvements from baseline to follow-up were having specific contact people for referral agencies (6 of 10 schools) and having instructions available for clinicians when making mandated reports to law enforcement (7 of 10 schools). Notably, there were two items in which no clinics reported attaining. Both related to IPV/SV data collection and feedback. One item was feedback on IPV/SV assessment or patient satisfaction surveys, and a second item was providing regular feedback to providers about IPV/SV assessment performance.

### Student-level data: exit survey

Proportion of students reporting whether their provider spoke with them about SV (intervention schools, median: 23, IQR: 11, 66; control schools, median: 6, IQR: 2, 10) or whether they disclosed SV during their visit varied widely by school (intervention schools, median: 8 IQR: 4, 31; control schools, median: 6 IQR: 3, 14). Unsurprisingly, students at intervention schools where providers received instruction and tools to facilitate SV discussions reported more discussions with providers, with seven intervention schools making up the top quartile. However, for both discussions (*n* = 2) and disclosures (*n* = 3), intervention schools were in the bottom quartile. While no control schools achieved numbers in the top quartile for discussing SV with students, four achieved the top quartile for students disclosing SV during their visit.

### Relationships between campus-, clinic-, and student-level data

When examining potential school level factors, only school size appeared to be associated with performance on campus-, clinic-, and student-reported metrics. Small schools (Table 2) appeared to be the highest performers in terms of student-reported data (i.e., students responding receiving SV discussion and card), while large schools tended to perform well on campus- and clinic-level measures. Specifically, the three schools that performed best in student-reported intervention implementation were small schools (Table 2, #5, 6, 8), while the three schools that performed worst were two large schools and one medium enrollment school (Table 2, #3, #7, #11).

QI data were similarly not always consistent with campus-level or student-reported data. For example, one large and one medium school (Table 2, #3 and #7), which both indicated high levels of campus engagement, knowledge, and policies also indicated higher scores on the QI tool at baseline (60 affirmative responses each). In contrast, their student-reported data revealed multiple areas in which these schools were among the bottom quartile. Another large school (Table 2, #10), with strong campus level findings, had lower quartile clinic-level findings, and with only 26 affirmative responses of a possible 89 and three improvements between baseline and follow-up on the QI tool.

**Fig. 1:**
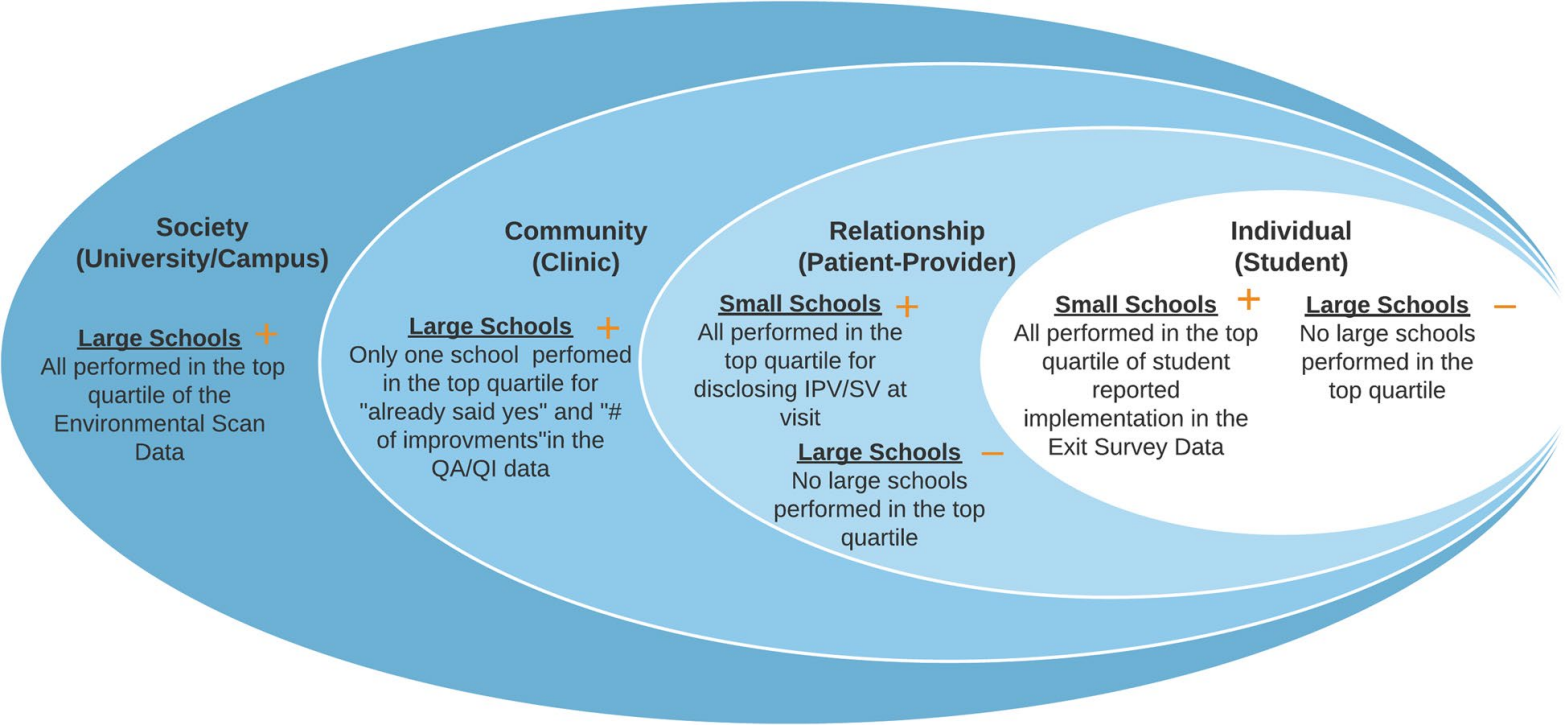
Socioeconomic model of results

Small schools, in both the intervention and control condition, dominated the top quartile of student-reported SV/IPV disclosure and discussion of SV/IPV. In our limited sample, this finding appears to cross urban and rural settings, religious and non-religious affiliated institutions, and campuses that reported all ranges of campus and clinic-level data (Fig. 1, Table 2).

## Discussion

Our results regarding the wide disparity between campus- and clinic-reported policies and procedures and student-reported interactions with providers demonstrates a key challenge for SV prevention and response on college campuses. Despite much attention to campus SV prevention in recent years, campus policies do not necessarily translate into on-the-ground actions.

Our study’s parent trial results found the intervention was effective in facilitating disclosure of violence and referral to resources when implemented as designed [16]. However, implementation varied widely and was not linked to performance on campus-level measures. Notably, schools that demonstrated high performance on campus or clinic-level data did not translate to intervention delivery. In many cases, we noted the opposite, with some of the highest performing schools (#3, #7), one large and one medium, delivering the intervention to only 8% of students during the study. Only one of the schools in the top quartile for environmental scan data delivered the intervention as intended to greater than 50% of students. Schools with health centers that performed best per student-reported data (#11, #8) fell in the bottom quartile of at least one of the climate measures yet delivered the intervention to more than 85% of students enrolled in the study.

Barriers to successful implementation of healthcare-based SV/IPV interventions have been previously evaluated. Consistent barriers reported by providers include lack of time, knowledge, and support [18–21]. Despite the resources of a clinical trial, data collected from providers noted similar barriers, and these barriers were often felt to be outside the control of the individual provider [22]. While our data collection methods do not link the provider data and the school level data presented here, other health systems’ literature supports that larger, often more well-resourced systems are those that are likely to have more complex systems and barriers to building individual patient-provider connections [23]. Larger campus settings, may be more likely to have electronic medical records, see higher patient volumes, manage more complex health conditions onsite rather than referring to off-campus providers, and face the burden of a financial bottom line—billing visits to students’ insurance rather than covering services as part of student fees (more common in smaller schools). Further investigation of these barriers may reveal other barriers, as well as opportunities for improvement.

The finding that small schools performed better on student-reported measures while large schools performed better on campus and clinic level measures merits additional explication. This finding highlights a significant gap between the student experiences of services compared to the administration’s perception of services [24, 25]. This indicates that “on paper” policies, at the campus and clinic level, may not be translated to meaningful changes in provider-patient interactions, highlighting the need for multi-level interventions to increase implementation of such clinic-based SV prevention interventions.

Potential reasons for this finding may include written policies not being fully implemented, variations in resources between campuses (primary campuses vs. branch campuses), and policies written in a way that protect schools’ legal interests but limits details on making services more student-centered and accessible [26–28]. For example, policies may detail formal reporting, investigative, and adjudication measure, but take control away from survivors rather than allotting equal time and space to confidential reporting and help seeking mechanisms [29]. Similarly, policies tying help for accommodations, medical care, and counseling to formal reporting can discourage students from seeking help [24, 25, 29]. Less than half of students seek any formal service following SV, and students report consequences of reporting are often worse than consequences of the initial assault [24, 25, 29]. Likewise, universities that proclaim to have “solved” SV on campus through policies and procedures captured in the environmental scan may discourage students and providers from engaging in meaningful discussion around their experiences with SV for fear of being deemed “system failures” or “acting outside the chain of command.”

Another concern is with translation of written policies from macro to micro environments. Having a written policy does not equal translation to practice. Individual provider knowledge and skills vary and may not always be sufficient to implement the policies as written [22, 30, 31]. Similarly, access to resources (e.g., time, private space, connections to SV/IPV advocates) is a potential reason for the variation in small compared to large school performance that we begin to note in this review of case data. Importantly, additional work is necessary to determine whether these conclusions maintain into other datasets or settings.

Importantly, our findings from this work have allowed us to begin to work with campus and community partners to continue addressing the barriers noted as most salient to implementation, sharing successes and strategies from other campuses as starting points, while having a broad picture of how vastly different the landscape is between these organizations. Our ongoing work aims to create more tailored and multilevel strategies for implementing the intervention across campuses to assess their impact on its adoption. Future clinical trials of behavioral interventions—in which high control over the environment, participants, and interventionists may not be feasible—may consider similar mixed efficacy-implementation methods to proactively collect and assess implementation data.

### Implications for public health practice

Given the short- and long-term negative consequences of SV, all levels of prevention and response remain of highest concern to public health professionals and campus administrators alike. Interventions are only successful if they reach their target audience. Understanding the context around how and why the intervention did not successfully reach students can help to inform future work with campuses in efforts to address this issue. This case-based analysis found some patterns between school size and performance. Small schools delivered the intervention to students more consistently, despite large schools reporting more formal SV policies, procedures, and resources.

Per-student resources (e.g., the number of health care providers per student) that may have allowed individuals in smaller schools to spend more time with patients in which relationships were established was unable to be observed. A possible benefit for student health and counseling centers in smaller schools may be their ability to operationalize them with less formality, therefore incorporating trauma-sensitive principles. This flexibility may include not requiring insurance for appointments, allowing students to walk in without set appointment times, and not having a cap on the number of counseling sessions students can have. Larger schools that service more students and rely on insurance reimbursement for funding instead of student fees may be less able to integrate changes that promote building trusting relationships between students and providers. Further exploration into opportunities to support providers is needed to ensure equitable implementation of this and other public health interventions based in student health settings. Possible avenues for exploration based on our findings and prior work include examination of alternate funding models (e.g., universal health care or medical home models).

### Limitations

As with any study, findings must be interpreted in the context of its limitations. Of note, we relied on a combination of publicly available data and campus administrator or staff-reported information to complete the environmental scans, while the QI tools relied solely on clinic administrator or staff-reported data. As such, there were both missing data and areas in which inconsistent data arose. For example, one institution had QI tool data in which the 18 items changed from “yes” to “no” during the 6-month implementation period. While it is not impossible for a clinic to have changes in policies that would create these responses, we question whether 18 is a true representation of changes, a difference in interpretation of the items, or lack of knowledge to answer the items (including the tool being completed by another clinic staff person).

Future use of the tools in research may minimize confusion regarding reporting by eliminating “not applicable” responses from items in which a response is indicated for all institutions and include options for “I don’t know” or “decline to respond” in order to capture a more complete picture of data from the individual answering. As the tool is designed for QI purposes, it is also most useful to institutions in which the users completing the tool have both access to the necessary information *and* a vested interest in improving the outcome. Lastly, while the data presented here allows us to importantly visualize and compare data from a variety of sources, the case series method does not allow for formal statistical testing, and generalizations from these findings must be done cautiously.

## Conclusion

In this case series, we did not see concordance between reported campus and clinic environments and the implementation of a health and counseling center-based SV intervention. When examining school level characteristics, small schools did appear to have more consistent intervention implementation and performed well among student-reported outcomes including student-reported disclosures of SV/IPV and receipt of resources or referrals. While ample evidence demonstrates that multilevel intervention strategies are necessary to address violence, our findings in this case series suggest that high level “on paper” policy changes are not sufficient to overcome barriers of consistently getting appropriate assessments, resources, referrals, and services to students in campus health and counseling centers.

## Supplementary Information

**Additional file 1.**

**Additional file 2.**

## Data Availability

The datasets used and/or analyzed during the current study are available from the corresponding author on reasonable request.

## Abbreviations

SV: Sexual violence
IPV: Intimate partner violence
QI: Quality improvement
IQR: Interquartile range

## Footnotes

1 ^*^Campus safety card has since been updated based on feedback obtained from students and providers. Current safety card is available for free download from: https://www.futureswithoutviolence.org/sex-relationships-and-respect-on-campus-safety-card/
