# Supplementary material for: A case series measuring campus and clinic level factors during implementation of a sexual violence prevention intervention in campus health and counseling centers: does environment matter?
Source: Implement Sci Commun. 2023 Jul 31;4:88. doi: 10.1186/s43058-023-00467-7 (PMC10388455; doi:10.1186/s43058-023-00467-7)
Supplement: Supplementary file 1 — Additional file 1. [file 43058_2023_467_MOESM1_ESM.docx]

| **College Health Center-based Alcohol and Sexual Violence Intervention Study**  **Quality Assessment/Quality Improvement Tool** |
| --- |
| Thank you for your participation in our study. This study aims to evaluate two different approaches to reducing alcohol-related violence on college campuses by training college health services in the administration of two different intervention techniques and analyzing the effectiveness of each one.  This tool is intended to provide campus health centers with some guiding questions to assess the quality of care related to 1) promotion of healthy relationships and consensual sexual activity; and 2) intervention related to dating/intimate partner and sexual violence (IPV/SV). The information can be used as a benchmark for each program to engage in quality improvement efforts, as well as a measurement tool for the study***. Complete the tool as honestly and completely as you can—there are no right or wrong answers***. For questions that you respond yes to, please attach the corresponding form, policy, tool, etc. Programs will complete the tool again about every 6 months.  We hope that this tool will help provide guidance on how to enhance your program’s IPV/SV prevention and response efforts. We will also be providing technical assistance, free materials, and training support as part of your program’s participation in the study.  Please let us know if you have any questions.  Rebecca Dick, MS Elizabeth Miller, PhD, MD  Study Coordinator Principal Investigator  [rebecca.dick@chp.edu](mailto:rebecca.dick@chp.edu) [elizabeth.miller@chp.edu](mailto:elizabeth.miller@chp.edu)  412-692-6581 412-692-8504 |
|  |

| **Completed by (title only)**: | | | | | |
| --- | --- | --- | --- | --- | --- |
| **College/University name:** | | | | | |
| **Date**: | | | | | |
| **Protocols** | | | | | |
|  | **Yes** | **No** | **N/A** | **Don't Know** | **Additional Comments** |
| **Does your health center have a written protocol for assessment^[[1]](#footnote-1)^ and response to Dating/Intimate Partner Violence (IPV)?** |  |  |  |  |  |
|  | **Yes** | **No** | **N/A** | **Don't Know** | **Additional Comments** |
| **Does your health center have a written protocol for assessment and response to Sexual Violence (SV)?** |  |  |  |  |  |
| **Are there sample wording, scripts, prompts, questions, or information on medical/health history/risk assessment forms or EHR for staff to:** | | | | | |
| Explain that every patient gets screened for IPV/SV because it is common and impacts health? |  |  |  |  |  |
| Inform patients about confidentiality and any mandated reporting requirements? |  |  |  |  |  |
| Ask patients about IPV (with sample questions)? |  |  |  |  |  |
| Ask patients about SV (with sample questions)? |  |  |  |  |  |
| Educate patients about impact of IPV/SV on health and wellbeing? |  |  |  |  |  |
| Discuss ways to stay safe in an unhealthy or abusive relationship? |  |  |  |  |  |
| Provide information about campus and community IPV resources? |  |  |  |  |  |
| **Do your protocols instruct providers to assess for IPV/SV during:** | | | | | |
|  | **Yes** | **No** | **N/A** | **Don't Know** |  |
| A visit addressing alcohol or other drug use |  |  |  |  |  |
| A visit addressing difficulty transitioning to college |  |  |  |  |  |
| A visit addressing weight/disordered eating/disordered exercise |  |  |  |  |  |
| A visit addressing grades/academic suspension |  |  |  |  |  |
| A visit addressing depression or suicidality |  |  |  |  |  |
| Any primary care visit |  |  |  |  |  |
| Any reproductive or sexual health visit |  |  |  |  |  |
| A wellness visit/Annual exam/Preventive Care |  |  |  |  |  |

| **Does your health center:** | | | | |
| --- | --- | --- | --- | --- |
|  | **Yes** | **No** | **N/A** | **Don't Know** |
| Provide patients with a written explanation of confidentiality and limits of confidentiality when they check-in? |  |  |  |  |
| Have a policy to ensure that providers ask about IPV/SV when the patient is alone? |  |  |  |  |
| Have a place to speak with clients privately? |  |  |  |  |
| Have a privacy screen on the computer to protect the contents of the electronic health record from being viewed by others? |  |  |  |  |
| **Assessment Methods** | | | | |
| **How are patients assessed for IPV/SV?** | | | | |
|  | **Yes** | **No** | **N/A** | **Don't Know** |
| Patients answer questions on a medical/health history form |  |  |  |  |
| Staff review the medical/ health history form and ask follow-up questions |  |  |  |  |
| Staff ask the patients questions |  |  |  |  |
| Staff to offer a palm-size safety card with information about how violence can impact health |  |  |  |  |
| Assessment occurs in a private place |  |  |  |  |
| **Which staff are primarily responsible for assessing patients for IPV/SV? (please pick one)**  Counselor/Therapist  Peer Health Educator  Medical Assistant  NP  RN  MD or DO  Other (Please explain) ___________________________ | | | | |
| **How often are patients asked about IPV/SV?**  Every time they come in for a visit  At initial visit  With each new sexual partner  At least every six months  At least once a year  No established time interval | | | | |

| **Documentation of Assessment and Response** | | | | | |
| --- | --- | --- | --- | --- | --- |
| **On the medical/health history/assessment form(s) are following steps documented?** | | | | | |
|  | **Yes** | | **No** | **N/A** | **Don't Know** |
| *Whose Got Your Back?* and/or other safety card was offered and discussed |  | |  |  |  |
| Harm reduction strategies were shared |  | |  |  |  |
| Referral to on campus resources and/or Title IX officer |  | |  |  |  |
| Referral to off campus resources a rape crisis center and/or domestic violence agency. |  | |  |  |  |
| **Intervention Strategies** | | | | | |
| **Does your staff:** | | | | | |
|  | **Yes** | | **No** | **N/A** | **Don't Know** |
| Have sample wording or scripts about what to say and do when a patient discloses IPV/SV? |  | |  |  |  |
| Have sample or scripted tools and instructions on how to do safety planning with patients who disclose current IPV/SV? |  | |  |  |  |
| Have instructions on how to file a mandated law enforcement report, including who to report to and what information must be collected? |  | |  |  |  |
| Have sample or scripted tools to provide a warm referral to IPV/SV agency? |  | |  |  |  |
| Have a safe place where the patient can use a phone at your health center to call a national hotline or to talk to a local rape crisis counselor &/or domestic violence advocate? |  | |  |  |  |
| Have instructions on campus reporting requirements, including who to report to and what information must be collected? |  | |  |  |  |
| **Do your staff have resource lists that:** | | | | | |
|  | | **Yes** | **No** | **N/A** | **Don't Know** |
| Identify referrals and resources such as Title IX office/campus resources, hotlines, support groups, shelters, legal advocacy, etc. for patients who disclose IPV/SV? | |  |  |  |  |

|  | **Yes** | | **No** | **N/A** | **Don't Know** |
| --- | --- | --- | --- | --- | --- |
| Identify referrals and resources for perpetrators of IPV/SV? |  | |  |  |  |
| Include a contact person for each referral agency? |  | |  |  |  |
| **Is there a staff person responsible for updating these lists?**  Yes  No  N/A  Don’t Know  If Yes, please include staff title/position: | | | | | |
| **Are these lists updated at least once a year?**  Yes  No  N/A  Don’t Know  If Yes, please describe the time interval: | | | | | |
| **Networking and Training** | | | | | |
| **Within the last year, has your staff had contact with representatives from any of the following campus departments and community-based agencies (contact means--called to refer a patient, called for assistance with a patient, called for information about program)?** | | | | | |
|  | | **Yes** | **No** | **N/A** | **Don't Know** |
| Campus-based domestic violence advocates | |  |  |  |  |
| Campus-based rape crisis counselor/CSART | |  |  |  |  |
| Campus based general counseling | |  |  |  |  |
| Off campus domestic violence advocates | |  |  |  |  |
| Off campus rape crisis counselor | |  |  |  |  |
| Campus safety office/campus police | |  |  |  |  |
| Local law enforcement | |  |  |  |  |
| Local hospital | |  |  |  |  |
| Campus Title IX Coordinator | |  |  |  |  |
| Dean of Students | |  |  |  |  |
| Residential Affairs | |  |  |  |  |
| Greek Affairs (fraternities and sororities) | |  |  |  |  |
| Peer Health Educators (prevention activities) | |  |  |  |  |

| **Are there any staff who are especially skilled/comfortable dealing with IPV/SV that other staff can turn to for help?**  Yes  No  If Yes, please include staff title/position: | | | | | |
| --- | --- | --- | --- | --- | --- |
| **Do your protocols advise staff on what to do if they do not feel comfortable or adequately skilled to help a patient when IPV/SV is disclosed? (Provide support for staff, where staff can go to get support, increase knowledge/capacity, etc.)**  Yes  No | | | | | |
| **Do any of your staff participate in a local IPV/SV task force or related subcommittee?**  Yes  No  If yes, please identify staff and describe task force/subcommittee: | | | | | |
| **Is there a buddy system or internal referral for staff to turn to for assistance when they are overwhelmed or uncomfortable addressing violence with a patient?**  Yes  No  If yes, please describe: | | | | | |
| **Within the last two years, have representatives from any of the following agencies/departments either been contacted to schedule a training or come to your health center and conducted a training for your staff?** | | | | | |
|  | | **Yes** | **No** | **N/A** | **Don't Know** |
| Campus-based domestic violence program |  | |  |  |  |
| Campus-based rape crisis center/CSART |  | |  |  |  |
| Campus based counseling services |  | |  |  |  |
| Off campus domestic violence program |  | |  |  |  |
| Off campus rape crisis center |  | |  |  |  |
| Campus safety/police | |  |  |  |  |
| Title IX Coordinator | |  |  |  |  |
| Peer Health Educators | |  |  |  |  |
| Other campus resources (list here): | |  |  |  |  |
| **What type of training(s) do new staff receive on IPV/SV?** | | | | | |

| **Self-Care and Support** | | | | |
| --- | --- | --- | --- | --- |
| **Does your health center:** | | | | |
|  | **Yes** | **No** | **N/A** | **Don't Know** |
| Have a protocol for what to do if a staff person is experiencing IPV/SV? |  |  |  |  |
| Have a protocol for what to do if a perpetrator is on-site and displaying threatening behaviors or trying to get information? |  |  |  |  |
| Provide individual clinical supervision for staff where they can discuss any concerns/ discomfort relating to IPV/SV cases? |  |  |  |  |
| Provide other types (group supervision, case presentation) of opportunities for staff to discuss any concerns/issues/etc relating to difficult cases? |  |  |  |  |
| Have an employee assistance program (EAP) that staff can access for help with current or past victimization? |  |  |  |  |
| **Data and Evaluation** | | | | |
| **Does your health center:** | | | | |
|  | **Yes** | **No** | **N/A** | **Don't Know** |
| Record the number of patients assessed for IPV? |  |  |  |  |
| Record the number of patients who disclose IPV? |  |  |  |  |
| Record the number of patients assessed for SV? |  |  |  |  |
| Record the number of patients who disclose SV? |  |  |  |  |
| Annually review all health center protocols relating to IPV/SV (both patient and staff related)? |  |  |  |  |
| Do any of your patient satisfaction surveys include any questions soliciting patient's opinions about assessment and intervention strategies for IPV/SV? |  |  |  |  |
| Provide regular (at least annual) feedback to providers about their performance regarding IPV/SV assessment? |  |  |  |  |
| **Education and Prevention** | | | | |
| **Does your health center:** | | | | |
|  | **Yes** | **No** | **N/A** | **Don't Know** |
| Provide information to patients on healthy relationships and consensual sexual activity? |  |  |  |  |
| Sponsor educational events (workshops, lectures, health fairs, etc.) to talk about healthy relationships and consensual sexual activity or indicators of abuse? |  |  |  |  |
| **Environment and Resources** | | | | |
| **Does your health center have any of the following?** | | | | |
|  | **Yes** | **No** | **N/A** | **Don't Know** |
| Brochures or information about IPV that patients can take |  |  |  |  |
| Brochures or information about SV that patients can take |  |  |  |  |
| Brochures, cards, information for patients about how violence exposure affects their health and well-being |  |  |  |  |
| Brochures, cards, information for patients about bystander/upstander intervention and primary prevention of IPV/SV |  |  |  |  |
| Brochures/cards/posters placed in an easily visible location |  |  |  |  |
| Has your health center adapted any materials to make them more culturally relevant for your patient population?  Yes  No  If yes, please describe: | | | | |
| Who is responsible for stocking and ordering health promotion materials such as brochures and posters?  Please identify staff by title: | | | | |
| **Additional Comments and Observations** | | | | |
|  | | | | |

1. *Throughout this document, we refer to assessment—rather than screening—for domestic violence. Screening refers to stand alone questions or a self-administered checklist, while assessment includes conversation with the provider that includes anticipatory guidance on healthy relationships, direct questions about IPV/SV, and harm reduction strategies and a warm referral to IPV/SV services if abuse is disclosed. [↑](#footnote-ref-1)
