# Supplementary material for: A case series measuring campus and clinic level factors during implementation of a sexual violence prevention intervention in campus health and counseling centers: does environment matter?
Source: Implement Sci Commun. 2023 Jul 31;4:88. doi: 10.1186/s43058-023-00467-7 (PMC10388455; doi:10.1186/s43058-023-00467-7)
Supplement: Supplementary file 2 — Additional file 2. [file 43058_2023_467_MOESM2_ESM.docx]

Directions: The following campus environmental scan is a tool to help you identify strengths and gaps in your sexual violence and intimate partner violence work on campus. Please fill in answer each question to the best of your knowledge. You are welcome to ask others for assistance to complete the scan.

| **Institution-wide Collaboration** | | |
| --- | --- | --- |
| At your institution, do individuals with different responsibilities for responding to sexual violence meet regularly as a team (such as a sexual violence task force) to coordinate their efforts? | | |
| Yes | No  *(skip to next section)* | Don’t Know  *(skip to next section)* |
| If so, who participates on your institution’s task force/other group? Check all that apply  Title IX Coordinator   Campus safety  Academic affairs Residential life Health services  Local rape crisis center  Students | | |
| How often does this team meet?  _______________________________________________________________________ | | |
| Who organizes this task force/other group and to whom do they report?  _______________________________________________________________________  _______________________________________________________________________  _______________________________________________________________________ | | |
| Is this team charged with reviewing the institution’s sexual misconduct policy or other related policy? | | |
| Yes | No | Don’t Know |
|  | | |
|  | | |

| **Data Collection and Evaluation** | | |
| --- | --- | --- |
| Is your institution’s Clery Act report easily accessible?  Yes 🡪 if it is accessible online, enter the website here:___  No | | |
| Please review your institution’s Clery Act report. For the most recent year, what is the total number of incidents in the following categories?   \| Category \| # of Incidents \| \| \| \| --- \| --- \| --- \| --- \| \| On-Campus* \| Non-Campus \| Public Property \| \| Aggravated Assault \|  \|  \|  \| \| Forcible Sex Offense \|  \|  \|  \| \| Non-Forcible Sex Offense \|  \|  \|  \| \| Dating Violence \|  \|  \|  \| \| Domestic Violence \|  \|  \|  \| \| Stalking \|  \|  \|  \| \| Hate/Bias Crimes – Sexual Orientation \|  \|  \|  \| \| Hate/Bias Crimes – Gender \|  \|  \|  \| \| Hate/Bias Crimes – All  (sum across all categories) \|  \|  \|  \|   **include Residential Facilities in this column*  What year are these data from (i.e. what is most recent year)? ______ | | |
|  | | |
| At your institution, who is responsible (title) for collecting and reporting Clery statistics? | | |
| Do you believe your institution’s reported Clery statistics accurately reflect the prevalence of sexual violence at your institution? | | |
| Has your institution conducted a campus climate survey in the past three years regarding sexual violence? | | |
| Yes  .  Website to view survey:*_______* | No    *(skip to next section)* | Don’t Know    *(skip to next section)* |
| Did the survey ask questions about students’ perceptions of specific locations on- or off-campus where they are particularly vulnerable to sexual assault?  Yes No Don’t Know | | |
| Did the survey ask questions about students’ perceptions of the effectiveness of the institution’s reporting options and investigation and adjudication proceedings?  Yes No Don’t Know    _______________________________________________________________________________  Did the survey ask questions about students’ perceptions of the adequacy of the institution’s accommodations and support services?  Yes No Don’t Know  Did the survey ask questions about the prevalence of sexual assault, dating violence, and stalking on campus? | | |
| Yes No Don’t Know | | |

| **Prevention and Education** | | | |
| --- | --- | --- | --- |
| Are incoming students required to complete a primary prevention* program on sexual violence? | | | |
| Yes | | No  *(skip next 3 questions)* | Don’t Know |
| ** Primary prevention involves stopping violence before it occurs; preventing initial victimization and perpetration of violence.* | | | |
| Who provides the primary prevention program? Please describe: _ | | | |
| Is the primary prevention program on sexual violence mandatory? | | | |
| Yes No Don’t Know | | | |
| How and when is the primary prevention program delivered?  Online and completed before coming to campus  In-person and completed during orientation  Both  Other (please describe) __________________________________________________  *(Request validation)* | | | |
| Are prevention education activities conducted throughout the institution year, and for all students? | | | |
| Yes | | No | Don’t Know |
| Does your institution’s prevention education activities include signs and posters? | | | |
| Yes | | No | Don’t Know |
| Does your institution’s prevention education activities include information in bathrooms? | | | |
| Yes | | No | Don’t Know |
| Does your institution’s prevention education activities include dorm-based programs? | | | |
| Yes | | No | Don’t Know |
| Does your institution’s prevention education activities include plays and skits? | | | |
| Yes | | No | Don’t Know |
| Does your institution’s prevention education activities include anything else? | | | |
| Yes | | No | Don’t Know |
| If so, what?  Does your institution evaluate the effectiveness of its prevention education programming, such as by conducting pre- and post- participation surveys, focus groups, or interviews? | | | |
|  | | | |

| **Reporting Process** | | |
| --- | --- | --- |
| In your opinion, does your institution have clear reporting options for students who experience sexual violence? | | |
| Yes | No | Don’t Know |
| Can students report sexual violence anonymously (allowing them to notify their institution of an incident without disclosing their name to the institution)? | | |
| Yes | No | Don’t Know |
| Based on your interactions with students, do you believe students generally understand:   1. Who to speak with if they wish to anonymously report sexual assault?   Yes No Don’t Know   1. Who will be obligated to report a student disclosure of sexual violence to the Title IX Coordinator?   Yes No Don’t Know | | |
|  | | |
| Under your institution’s policy, who is required to report incidents of sexual assault, dating violence, domestic violence, and stalking to the Title IX Coordinator if a student survivor discloses an incident to them?  _______________________________________________________________________  _______________________________________________________________________  _______________________________________________________________________ | | |

| **Investigation and Adjudication** | | |
| --- | --- | --- |
| Is there a specific person who is responsible for explaining the option of pursuing an institution investigation and adjudication to a student who has experienced sexual violence? | | |
| Yes | No | Don’t Know |
| Does your institution have a full-time Title IX Coordinator whose job duties are exclusively focused on compliance with Title IX and related matters?  Yes No Don’t Know    *(skip next question)* | | |

| If the Title IX Coordinator at your institution has additional responsibilities besides Title IX matters, what are the Title IX Coordinator’s additional responsibilities? |
| --- |
| _______________________________________________________________________  _____________________________________________________________________ |
| What training does the Title IX Coordinator receive? |
| _______________________________________________________________________ _______________________________________________________________________ _______________________________________________________________________ |
| Who conducts investigations of sexual misconduct at your institution?  ____  _______________________________________________________________________ |
| Is there a Memorandum of Understanding between your institution and local law enforcement regarding sexual violence investigations?  Yes No Don’t Know |
| How is an accused student informed of a sexual assault complaint against him/her/them, and informed of the investigation and adjudication process?  Conduct officer will set up meeting with the referring student and the accused student_______________________________________________________________________  Does your institution provide the complainant and accused student with expected timeframes in which it will complete an investigation?  Yes  If yes, what are they?  What referrals (if any) are made for accused students?  What is done to follow through or monitor after the referral is made? |

| **Survivor Support Services & Accommodations** | | |
| --- | --- | --- |
| Does your institution have 24/7 crises and response for survivors of sexual violence?  *(force validation)*  Yes No Don’t Know  *(skip to SANE question)* | | |
| If yes, please answer the following questions about how 24/7 crisis and response is provided: | | |
| Who provides 24/7 crises and response? | | |
| Is there a 24/7 crisis-response line?  Yes No  If yes, please list the number(s): | | |
| If there is a request for in-person response, who shows up? _____________________ | | |
| If medical attention is needed, where are services provided? _______________________  Is this medical care available 24/7?  Yes No | | |
| If counseling is needed, where are services provided? ___________________________  Is this counseling care available 24/7?  Yes No | | |
| Will your institution provide a student with transportation to off-campus medical and/or counseling services?  Yes No | | |
| Is there a nearby hospital with a SANE program where students can be referred?  Yes No  If yes, in your opinion, do students know about it?  Yes No | | |
| Is your institution’s student health center trained to provide a trauma-informed response to survivors of sexual violence? | | |
| Yes  If yes, who trained the staff and when?_____________ | No | Don’t Know |
| Can students who experience sexual violence obtain accommodations and other support services at the health center after experiencing sexual violence without making a report of the incident to the institution?  Yes No Don’t Know | | |
| Are housing transfers available as an accommodation for survivors? | | |
| Yes | No | Don’t Know |
| Are class transfers available as an accommodation for survivors? | | |
| Yes | No | Don’t Know |
| Are extensions of time for classwork available as an accommodation for survivors?  Yes No Don’t Know | | |
| Are grade changes available as an accommodation for survivors? | | |
| Yes | No | Don’t Know |

| Are other accommodations available to survivors? | | |
| --- | --- | --- |
| Yes | No | Don’t Know |
| What are they? ___________________________________________________________ _______________________________________________________________________ _______________________________________________________________________ _______________________________________________________________________ | | |
| Has your institution ever expelled a student for sexual misconduct? | | |
| Yes | No | Don’t Know |
| Does your institution make transcript notations to indicate that a student withdrew while under investigation for sexual misconduct or was found responsible for sexual misconduct?  Does your institution evaluate the effectiveness of accommodations and response services offered to survivors?  Does your institution have any independent contracts/Memorandums of Understanding with sexual assault services outside of campus resources? | | |
| Yes | No | Don’t Know |
|  |  |  |
| If yes, what are they? _____________________________________________________ _______________________________________________________________________ _______________________________________________________________________ | | |
| **Workplace Policy** | | |
| Does your institution have a policy that addresses rights and options for employee survivors of sexual violence? | | |
| Yes | No | Don’t Know |
| Does your institution have a policy that prohibits employees from committing sexual violence?  Yes No Don’t Know | | |
| Does your institution have a policy that addresses procedures for reporting, investigating, and adjudicating incidents of sexual violence between employees and students? | | |
| Yes | No | Don’t Know |
| Does your institution have a policy that addresses procedures for reporting, investigating, and adjudicating incidents of sexual violence between administrators and staff members, and between professors and staff members? | | |
| Yes | No | Don’t Know |
| Does your institution have a policy that prohibits sexual and romantic relationships between professors and undergraduate students? | | |
| Yes | No | Don’t Know |

| Other information:  *Please use this space to provide any other information you feel is relevant.* |
| --- |

Who completed this Campus Environmental Scan (name & title)? ______________

Did you complete it alone or with assistance?

Alone

With assistance 🡪 who helped (title)? _____________

Date completed: _______________

Which institution?

California University of Pennsylvania

Carlow University

Carnegie Mellon University

Chatham University

Clarion University

Duquesne University

Edinboro University

Fairmont State University

Indiana University of Pennsylvania

La Roche College

Point Park University

PSU – Altoona

PSU Fayette

Robert Morris University

Saint Francis College

Slippery Rock University

UPitt – Bradford

UPitt – Johnstown

UPitt – Greensburg

UPitt – Pittsburgh

UPitt – Titusville

Washington & Jefferson College

West Virginia University

Westminster College
